# MIAME/Plant – adding value to plant microarrray experiments

**DOI:** 10.1186/1746-4811-2-1

**Published:** 2006-01-09

**Authors:** Philip Zimmermann, Beatrice Schildknecht, David Craigon, Margarita Garcia-Hernandez, Wilhelm Gruissem, Sean May, Gaurab Mukherjee, Helen Parkinson, Seung Rhee, Ulrich Wagner, Lars Hennig

**Affiliations:** 1 Swiss Federal Institute of Technology – ETH Zurich, ETH Center, CH-8092 Zurich, Switzerland; 2 The Nottingham Arabidopsis Stock Center (NASC), Division of Plant Sciences, University of Nottingham, Sutton Bonington LE12 5RD, UK; 3 The Arabidopsis Information Resource (TAIR), Carnegie Institution of Washington, 260 Panama St, Stanford, CA 94305, USA; 4 European Bioinformatics Institute (EBI), European Bioinformatics Institute, EMBL outstation, Wellcome Trust Genome Campus, Hinxton, Cambridge CB10 1SD, UK

## Abstract

Appropriate biological interpretation of microarray data calls for relevant experimental annotation. The widely accepted MIAME guidelines provide a generic, organism-independant standard for minimal information about microarray experiments. In its overall structure, MIAME is very general and specifications cover mostly technical aspects, while relevant organism-specific information useful to understand the underlying experiments is largely missing. If plant biologists want to use results from published microarray experiments, they need detailed information about biological aspects, such as growth conditions, harvesting time or harvested organ(s). Here, we propose MIAME/Plant, a standard describing which biological details to be captured for describing microarray experiments involving plants. We expect that a more detailed and more systematic annotation of microarray experiments will greatly increase the use of transcriptome data sets for the scientific community. The power and value of systematic annotation of microarray data is convincingly demonstrated by data warehouses such as Genevestigator^® ^or NASCArrays, and better experimental annotation will make these applications even more powerful.

High-throughput technologies are rapidly reshaping the plant research landscape. Gene expression microarrays, in particular, have impacted our way of thinking and challenged our concepts about research by providing genome-wide results at an increasing rate. A recent search in PubMed with the terms "microarray" and "plant" yielded more than 600 references, most of which report results obtained using RNA profiling technology. A major portion of the published datasets can be obtained via repositories, as supplements to scientific publications, or from the authors directly. Precise experiment annotation, however, that is easily accessible and suitable for comparisons, reproduction and/or correct interpretation of experiments is not always fully available. In fact, standard terms to describe conditions for plant microarray experiments so far have not been defined. While the technical details of a microarray experiment are usually abundant, the biological details are frequently poorly described, unsystematic between laboratories, or even missing.

MIAME (Minimum Information About a Microarray Experiment) is a standard that provides a conceptual framework for core information to be captured from most microarray experiments [1]. The microarray community has been very favorable to the establishment and implementation of this standard. Manufacturers, software developers and international databases have contributed to the development and adoption of the MIAME guidelines. Many scientists reporting microarray experiments now use this standard, which is required by an increasing number of scientific journals.

The MIAME standard has proven very useful so far. Data from repositories can be grouped according to standardized categories with respect to technology platform, labeling and hybridization procedures, measurement data, and array design. Nevertheless, experiment descriptions remained unsatisfactory for many researchers browsing databases, because the use of a common denominator for all biological sciences makes a full annotation of specialized community-level research difficult.

Specifically, the core MIAME standard is limited in its ability to capture domain-specific information on experimental design and sample preparation. Readers of publications describing plant microarray experiments are not only interested in hybridization or normalization protocols, but also e.g. whether the plants were grown under high or low light conditions, on soil or in vitro, or which organs where harvested at what age etc. Domain-specific extensions of the existing general MIAME standard are needed to collect experimental annotation in a structured way. MIAME/Plant, MIAME/Env and MIAME/Tox are such extensions [2-4].

In plant science, the use of benchmark terminology for growth stages [5] and plant organs [6] so far has allowed the pioneering development of powerful tools for data storage and analysis [7-9]. The rapidly increasing number of microarray studies on plants, together with the prospect of effective data querying by annotation of experimental parameters using controlled vocabularies, now requires a plant-specific MIAME standard. The objective of MIAME/Plant is to facilitate and normalize experiment and sample annotations (Figure 1). This will make annotation browsing and data analysis much easier for end-users, including experimental biologists. More specifically, MIAME/Plant incorporates the core MIAME standard together with a supplementary guideline for experimental description and design and sample preparation protocols (see Figures 1 and 2 for more details). The current complete MIAME/Plant guidelines can be downloaded from the Microarray Gene Expression Data Society, from the Nottingham Arabidopsis Stock Center and from The Arabidopsis Information Resource websites [10-12]. Currently, MIAME/Plant is being implemented in international repositories [13], and user-friendly tools will further encourage the establishment of these guidelines in the plant community.

**Figure 1:**
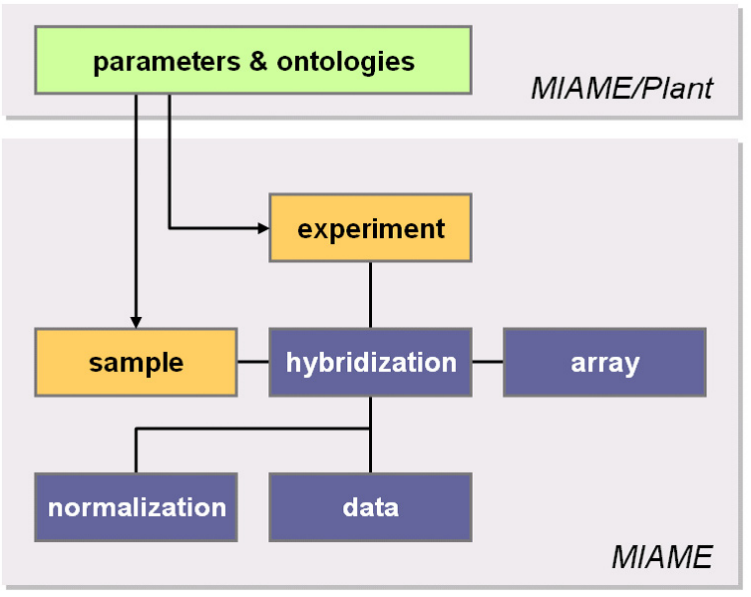
A schematic representation of the six components of a microarray experiment as defined by MIAME (Brazma et al., 2001). The MIAME/Plant parameters and ontologies extend the basic experiment and sample annotations.

**Figure 2:**
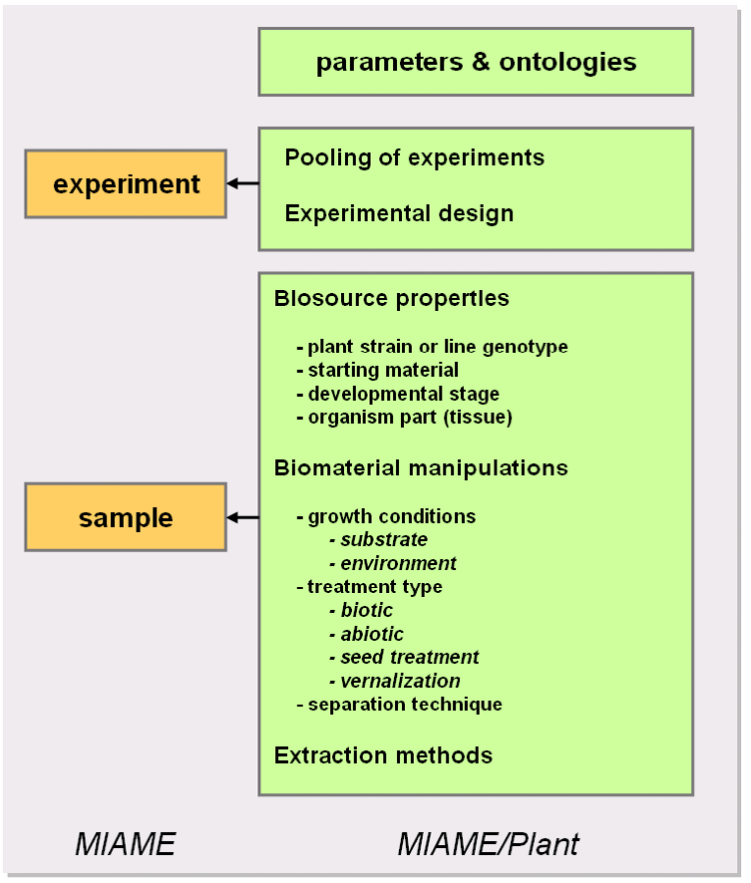
Overview of the main classes of ontologies currently represented in MIAME/Plant.

The implementation of MIAME/Plant should not create an extra burden on the experimenter, but will enable a targeted workflow through the annotation process that is facilitated by structured data entry forms. While all reported experiments should adhere to MIAME, we do not propose to enforce that every experiment should be annotated to the full detail of MIAME/Plant. Instead, laboratories that decide to collect additional information are encouraged to follow the MIAME/Plant scheme. Systematic and controlled annotation will generate breakthroughs in data mining capabilities, and therefore we encourage the plant community to adopt the MIAME/Plant standard. We expect that the benefits of domain-specific MIAME extensions will also motivate researchers in other fields to develop better community standards.
